# Pulp Fines—Characterization, Sheet Formation, and Comparison to Microfibrillated Cellulose

**DOI:** 10.3390/polym9080366

**Published:** 2017-08-17

**Authors:** Wolfgang Johann Fischer, Melanie Mayr, Stefan Spirk, David Reishofer, Lukas Andreas Jagiello, Romana Schmiedt, Jerome Colson, Armin Zankel, Wolfgang Bauer

**Affiliations:** 1 Institute of Paper, Pulp and Fibre Technology, Graz University of Technology, Inffeldgasse 23, 8010 Graz, Austria; WolfgangJohann.Fischer@sappi.com (W.J.F.); melanie.mayr@tugraz.at (M.M.); stefan.spirk@tugraz.at (S.S.); david.reishofer@tugraz.at (D.R.); l.jagiello@zellstoff-poels.at (L.A.J.); office.ipz@tugraz.at (R.S.); 2 Sappi Paper GmbH, Brucker Str. 21, 8101 Gratkorn, Austria; 3 Zellstoff Pöls AG, Doktor-Luigi-Angeli-Straße 9, 8761 Pöls, Austria; 4 Department of Materials Sciences and Process Engineering, Institute of Wood Technology and Renewable Materials, University of Natural Resources and Life Sciences Vienna, Konrad-Lorenz-Straße 24, 3430 Tulln, Austria; jerome.colson@boku.ac.at; 5 Institute for Electron Microscopy and Nanoanalysis, NAWI Graz, Graz University of Technology and Centre for Electron Microscopy, 8010 Graz, Austria; armin.zankel@felmi-zfe.at

**Keywords:** pulp fines, fiber fines, microfibrillated cellulose, sheet forming, vacuum filtration, tensile properties, contact angle, surface roughness

## Abstract

In the pulp and paper industry different types of pulp or fiber fines are generated during the pulping (primary fines, mechanical fines), and/or the refining process (secondary fines). Besides fibers, these cellulosic microparticles are a further component of the paper network. Fines, which are defined as the fraction of pulp that is able to pass through a mesh screen or a perforated plate having a hole diameter of 76 μm, are known to influence the properties of the final paper product. To better understand the effect and properties of this material, fines have to be separated from the pulp and investigated as an independent material. In the present study, fines are isolated from the pulp fraction by means of a laboratory pressure screen. To allow for further processing, the solids content of the produced fines suspension was increased using dissolved air flotation. Morphological properties of different types of fines and other cellulosic microparticles, such as microfibrillated celluloses (MFC) are determined and compared to each other. Furthermore, handsheets are prepared from these materials and properties, such as apparent density, contact angle, modulus of elasticity, and strain are measured giving similar results for the analyzed types of fines in comparison to the tested MFC grades. The analysis of the properties of fiber fines contributes on the one hand to a better understanding of how these materials influences the final paper products, and on the other hand, helps in identifying other potential applications of this material.

## 1. Introduction

Paper is a non-woven fibrous cellulose based material, which is formed by an interaction of individual fibers to form a network with a complex hierarchy and porosity. The large fraction of this network consists of pulp fibers, which have been subjected to a variety of treatments depending on the envisaged application for the final paper products. During these treatments in pulping and papermaking processes, cellulosic microparticles are formed, the so-called fines. Fines are defined as the fine cellulosic particles, which are able to pass through a 200 mesh screen (equivalent hole diameter 76 μm) of a conventional laboratory fractionation device (SCAN-CM 66:05). There are two major types of fiber fines, namely primary and secondary fines. Primary fines are generated during pulping and bleaching, where they are removed from the cell wall matrix by harsh chemical and mechanical treatment. As a consequence of their origin (i.e., compound middle lamella, ray cells, parenchyma cells), primary fines exhibit a flake-like structure with only minor shares of fibrillar material [1,2]. In contrast, secondary fines are generated during the refining of pulp. In this case, a mechanical treatment partially induces fibrillation of cell wall fractions, resulting in the formation of a fibrillated material that has microscopic dimensions [3,4]. Both types of fines are usually present in a papermaking furnish. However, due to their differences in morphology and chemistry, which is also dependent on the type of the pulping process, they have a different impact on paper quality and product performance [2,5,6]. Particularly the large aspect ratio, surface area, and bonding ability of secondary fines enhance the mechanical strength of papers, while having a negative influence on dewatering in the forming section of a paper machine. Primary fines mainly affect the optical properties of the final papers [2,7,8], but also have a negative effect on dewatering, and in some cases also on mechanical paper properties. Because of their large specific surface area in comparison to pulp fibers, fines also consume a high proportion of chemical additives used in pulp and paper production.

Fines usually are an inevitable, but undefined part of papermaking furnishes. Although in principle their effects on paper properties, being it sheet densification, strength improvement, deterioration of dewatering, etc. are known, their impact is often difficult to quantify. The partly negative effects of fines on process performance and paper properties are also a motivation to consider the removal of fines from papermaking furnishes.

There are two major issues when it comes to the fines fraction in papers. The first is related to the analysis of the distribution of the types of fines in papermaking furnishes and in paper, which is not a straightforward task [9]. The second is related to the isolation of the fines from paper pulps, which involves laborious separation steps (e.g., screening, centrifugation, etc.), resulting in suspensions with dry weight contents of 0.1 wt % and below [10,11,12,13,14]. Consequently, the removal of water to obtain higher dry weight contents is a major challenge. In contrast to microfibrillated celluloses (MFCs), which recently entered into the market, pulp fines so far are not commercially available. This is certainly associated with the above-mentioned problems in analysis and production/separation, as well as a lack of detailed investigations on their properties.

In the production of MFCs, various kinds of mechanical treatments are applied, such as high pressure homogenization, microfluidization, grinding, cryo-crushing, steamexplosion, and high intensity ultrasonfication, as summarized in a review by Osong et al. [15]. All these techniques require a significantly higher energy input of up to 30,000 kWh/t when compared to conventional refining [15]. Depending on the applied technique a more or less homogeneous MFC material is produced. In a microfluidizer, for example, the material has to undergo several homogenization passes to achieve a homogeneous fibrillation. Fibrillation is improved by chemical or enzymatical pretreatment to reduce energy consumption [16], but pretreatment and multiple passes are a cost factor in MFC production. In conventional refining, fibrillar (secondary) fines are produced at much lower energy levels than in MFC production. These fines, however, have to be separated from the remaining fibers in order to use it as a MFC substitute. Microfribrillation is directly related to the gap-clearance of the refiners, which is rather high in conventional refining when compared to super grinders, and thus tends to produce a coarser material. MFC is defined to have a length between 0.5 to 100 µm, and a width from 10 to 100 nm [15]. Since fines are defined as the particles passing through a 200 mesh screen, they contain also larger particles. For example, primary fines having a flake-like structure show dimensions of up to 350 µm × 25 µm in the fines fraction [17].

In this paper, we attempt to address the challenge of fines separation by first introducing an elegant method to upscale the production/separation method for fines to kg scale, then we investigate the sheet formation using different types of fines, and finally compare their performance to commercially available MFC products.

## 2. Materials and Methods

All tests have been performed using primary and secondary fines, separated from never-dried, unbleached softwood kraft pulp (a mixture of spruce and pine), never-dried, bleached sulfite pulp (spruce), as well as mechanical pulp fines from never-dried, bleached pressure ground wood (spruce). Furthermore, two commercially available microfibrillated cellulose samples, and one experimental MFC grade from an institution, that does not want to be disclosed, were used. The analyzed samples are summarized in Table 1.

### 2.1. Separation and Thickening of Fines

**Figure 1:**
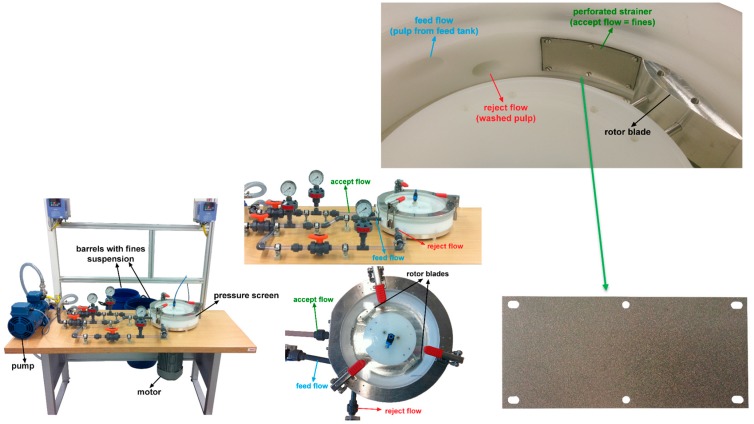
Pressure screen for separating fines from pulp.

In order to separate the fines from the pulp, a purpose-built laboratory pressure screen was employed. This newly designed device is capable of separating fines from pulps in kilogram scale [10]. Figure 1 depicts the pressure screen used in the present study.

**Figure 2:**
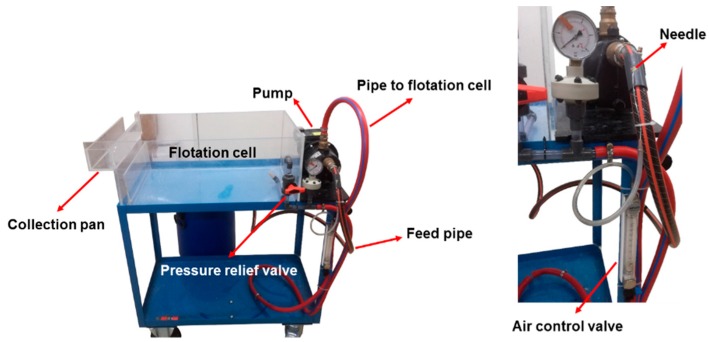
Flotation cell for thickening of fines (left); magnification of the air injection system.

In the first step of fines separation, the pulp was diluted with water to a consistency of 0.5 to 1 wt %, and stirred for about 10 min. This suspension was pumped through the pressure screen (PS), whose main element is a perforated strainer with hole diameters of 100 μm. Larger particles will not pass the strainer, and are transferred back to the feed tank. The fines fraction that is able to pass the strainer is collected in a separate barrel. Due to the low solid content of the fines suspension (0.02%,) a subsequent thickening step is needed. For this task, a laboratory flotation cell, which can be directly connected to the PS, is implemented. The flotation cell developed at the Institute of Paper, Pulp and Fibre Technology (Graz, Austria) is shown in Figure 2.

The fines suspension is pumped from the feed barrel to the flotation cell. Air is injected into the suspension with a needle positioned directly before the pump (see Figure 2, magnification right). The air flow can be adjusted by an air control valve. Shear forces inside the pump lead to the collapse of the air bubbles, and finally to the dissolution of air. Due to a pressure drop, controlled with a pressure relief valve, the air is released in the form of fine bubbles (Ø ~ 60 μm). The fines, attached to the air bubbles, rise to the surface of the water layer in the flotation cell and can be collected in the collection pan. Using this method, the solid content can be increased by up to 4% [10]. In contrast to other flotation methods, a major advantage here is to avoid the use of any chemicals in the course of the applied flotation process.

### 2.2. Lignin Content and Morphological Properties of Fines and MFC

For each of the materials, the κ-number, as an indication for the lignin content of a certain pulp or pulp fraction, was determined according to ISO 302:2015. An approximation of the mass fraction of lignin can be obtained by multiplying the κ-number by 0.15 [18].

The morphological properties of the fines, as well as of the microfibrillated cellulose are determined using the L & W Fiber Tester Plus (ABB, Mannheim, Germany). Morphological features of even small cellulosic microparticles can be detected (resolution: 3.3 μm/pixel). The size measurement was carried out on the fines fraction obtained using the pressure screen, MFC samples were measured without fractionation. No size definition criteria of the instrument were applied. For each test, a dry sample (0.1 g) is diluted with 150 mL of deionized water. A minimum of 100.000 particles was detected. For each sample, two replicates have been performed. The results of these measurements are expressed as the average circle equivalent diameter (CED_q^2^_, Equation (1)), which in terms of irregularly formed particles, can be used instead of the length weighted average particle length [4]. The CED is the diameter of a circle CED_i_ of equivalent area than the projection area A_i_ of the particle (Equation (2)). For an illustration of the differences between these samples additional images using a conventional transmission light microscope (Leica 301–371.010) were captured. Details regarding this method are described in [4].

(1) $${\bar{CED}}_{q^{2}}=\frac{CED_{i}^{3}}{CED_{i}^{2}}$$

(2) $$CED_{i}=\sqrt{\frac{4\times A_{i}}{\Pi }}$$

### 2.3. Fines and MFC Sheet Formation

**Figure 3:**
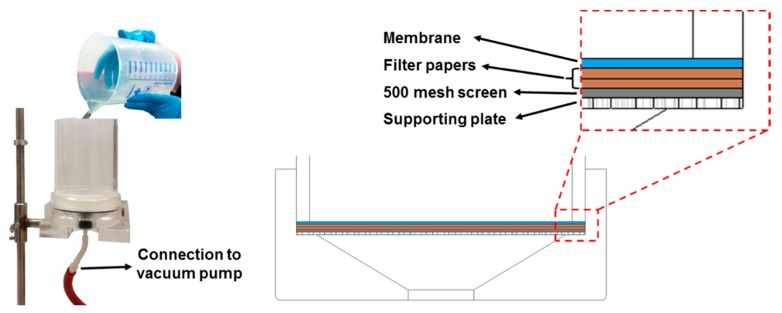
Equipment used for fines and MFC sheet formation.

In order to prepare handsheets from pure fines and MFC, a vacuum filtration method is used. A defined amount of fines or MFC (0.24 g, dry weight) is diluted with deionized water to reach a solid content of 0.2 wt %. This suspension is stirred at 450 rpm for at least 2 h. After stirring, the sheets are formed by vacuum filtration using a Britt Dynamic Drainage Jar (Frank PTI, Birkenau, Germany). The setup used for sheet forming is shown in Figure 3.

The Britt Dynamic Drainage Jar is equipped with a supporting plate, a 500-mesh screen (hole diameter 20 μm), two filter papers, and a nitrocellulose membrane (DAWP29325 from Merck Chemicals and Life Science GesmbH, Darmstadt, Germany) with a pore size of 0.65 μm. The major advantage of using the Britt Dynamic Drainage Jar as compared to a Büchner funnel is to improve the fines and MFC sheet formation. In particular, the sandwich-like setup prevents the loss of fine cellulosic material. After the filtration step, the membrane with the fines/MFC sheet on top is pre-dried in a Rapid-Köthen sheet dryer (Frank PTI, Birkenau, Germany) for about 20 s at 93 °C under vacuum. Then, the membrane is peeled off, and the neat fines/MFC sheets are dried for 10 min in the Rapid-Köthen sheet dryer. The sheets are then stored in a climate room at 23 °C and 50% RH for least 12 h prior to testing.

### 2.4. Sheet Testing

Air permeability (Bendtsen method, ISO 5636-3:2013), thickness, and apparent density (DIN EN ISO 534:2011) were determined in order to get structural information of the sheets. Mechanical properties such as the modulus of elasticity, breaking load, and strain to failure of the sheets were measured using the Z010 tensile tester from Zwick Roell (Ulm, Germany). For tensile testing, a testing speed of 5 mm/min, a free clamping length of 50 mm, and strip width of 15 mm were used; eight to ten strips per sample were tested.

The surface roughness of the sheets was determined using the Bruker DekTak XT surface profiler (Bruker Nano Surfaces Division, Tucson, AZ, USA). The needle scanning the sheets had a tip radius of 12.5 μm, and a scan force of 0.03 mN. The length of the line scan was set to 2 mm. 

For the atomic force microscopy (AFM) measurements, a Veeco Multimode Quadrax MM AFM (Bruker; Billerica, MA, USA) was used. The images were recorded in the tapping mode (non-contact mode), and silicon cantilevers (NCH-VS1-W, NanoWorld AG, Neuchatel, Switzerland) were used with an average spring constant of 42 N/m (Force Constant), and a resonance frequency of 281–296 kHz (Coating: none). All measurements were performed at room temperature and under an ambient atmosphere. In addition, the surface of the handsheets was investigated using low voltage scanning electron microscopy (LVSEM) with the high resolution scanning electron microscope Zeiss Ultra 55 (Zeiss, Oberkochen, Germany), and the Everhart–Thornley detector (ETD) [19]. A beam energy of 0.65 keV was used.

In order to determine the static contact angle, as well as the surface free energy of the sheets, a DSA100 (Krüss GmbH, Hamburg, Germany) equipped with a T1E CCD camera (Newton, NJ, USA) and the DSA1 v 1.90 software (Krüss GmbH, Hamburg, Germany) was used. 3 μL droplets of Milli-Q water and diiodomethane were deposited on the surface of the sheets, and after 2 s an image was taken. Ten replicates were performed. The contact angle was calculated according to the Young-Laplace equation. The surface free energy was calculated according to the Owens-Wendt-Rabel-Kaelble (OWRK) method.

## 3. Results and Discussion

### 3.1. Lignin Content and Fines/MFC Morphology

The results of the κ number measurements are shown in Table 2. As expected, fines from the unbleached pulp, as well as from the mechanical pulp show higher lignin contents when compared to the other materials. The high lignin content in the case of mechanical pulp fines is a result of the production process. In contrast to chemical pulping, the fibers in this case are separated from the wood matrix itself by a mechanical process with no removal of lignin. Furthermore, primary fines from unbleached kraft pulp, and to some extent also from bleached sulfite pulp have a higher κ number when compared to the secondary fines since they are derived from domains rich in lignin (e.g., compound middle lamella).

The mean particle size (see Table 3), expressed as CED_q^2^_ is similar for all the samples. Secondary fines, as well as the microfibrillated cellulose samples MFC 2 and MFC 3 show the smallest particle size. Some caution is needed regarding these results, as the resolution of the used L & W instrument might lead to the effect that particles in the sub-micron range just are not detected. Primary fines from unbleached kraft pulp and MFC 1 contain the largest particles. From the results, one might conclude that the pulp fines, especially secondary fines, and MFC, can be compared in terms of particle size, even though the two MFC samples (MFC 1 and MFC 3) have been prepared using homogenizers, when compared to the refining of pulp to produce secondary fines using a Valley beater. Taipale et al. [20] also already pointed out that the fines fraction of pulps exhibit properties similar to MFC.

**Figure 4:**
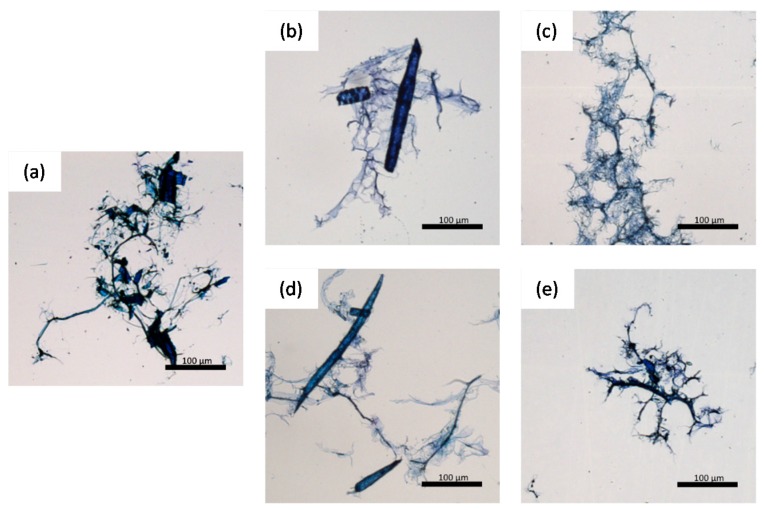
Optical microscopy images of (**a**) MP fines; (**b**) UB PF kraft pulp; (**c**) UB SF kraft pulp; (**d**) B PF sulfite pulp; (**e**) B SF sulfite pulp.

Optical microscopy images of pulp fines (Figure 4) reveal that size measurement is not straight forward since smaller fragments of microfibrillar fines might not be detected by the L & W Fiber Tester, due to its limited resolution of 3.3 µm/pixel [4]. Therefore, these images contribute to a better understanding regarding differences in fines morphology of the different materials. MP fines (a) contain fragments of the fiber wall and fibrillar material. However, these fibrils are more or less isolated, and do not form a network like structure. In contrast, chemical pulp fines (b–e) form fibrillar networks with a hierarchical, branched morphology. In addition to fibrillar fines originating from the fiber wall, primary fines (b,d) also contain rod shaped parenchyma and ray cells. These cells were fragmented from the wood matrix in the pulping process.

### 3.2. Results Sheet Testing

#### 3.2.1. Apparent Density of the Sheets

**Figure 5:**
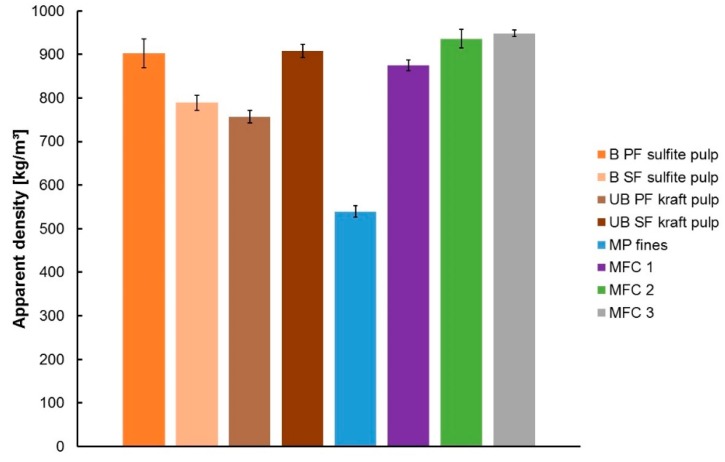
Apparent density of fines and MFC sheets.

Figure 5 summarizes the results of the apparent density measurements of the different sheets. The materials can be classified into three regimes of density. The lowest apparent density is determined for the fines sheets derived from mechanical pulp (ca. 500 kg·m^−3^). Mechanical pulp fines are known to be stiffer, thereby showing a lower conformability, which probably originates from the higher lignin content. Furthermore, the bonding ability of this material is lower, resulting in a potentially more porous sheet structure. The second set of samples comprises of the sheets made from B SF sulfite, and the UB PF kraft pulps (ca. 700–750 kg·m^−3^). The third set includes the B PF sulfite, the UB SF kraft sheets, as well as the different MFC sheets (900–950 kg·m^−3^). Regarding the sulfite pulps, sheets from primary fines are denser than those made from secondary fines. In contrast, for the unbleached kraft pulps, the secondary fines lead to denser sheets. These results could be qualitatively explained by the different morphologies of the samples. Samples with a higher degree of coarse graining (B SF sulfite and UP PF kraft) have a low density, while the other feature higher densities. In addition, the high density of PF sulfite might be reasoned by the thinner cell walls when compared to PF kraft and differences in chemical composition as well.

Apparent density data, however, have to be read with care. According to ISO 534:2011, apparent sheet density is determined by using a dead-weight micrometer, provided with two plane, parallel, circular pressure faces, with one face having a diameter of 16.5 mm, between which the sheet is placed for measurement. Therefore, the roughness of the sheet also influences the thickness measurement, and thus also the calculation of the apparent sheet density. Compressibility of the sheets also might influence the thickness measurement, as the pressure exerted between the two pressure faces during measurement is 50 kPa.

Air permeability (Bendtsen method) was 0 mL/min for all the sheets, with the exception of the MP fines sheets (15.5 mL/min), indicating that the sheets are closed.

#### 3.2.2. Surface Morphology and Roughness of the Sheets

**Figure 6:**
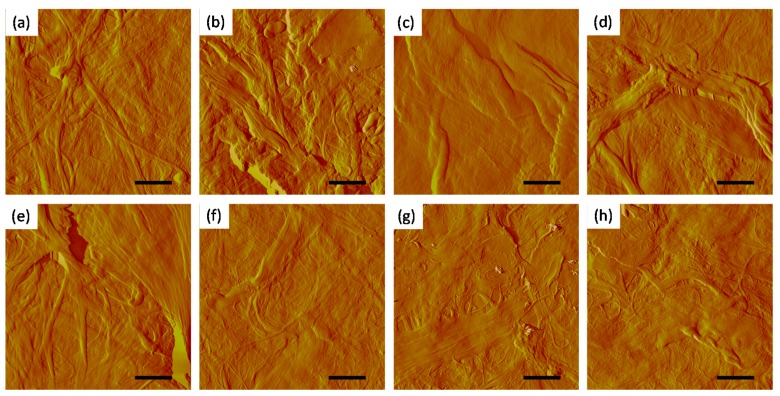
AFM amplitude images (10 × 10 µm^2^, scale bar = 2 µm) of sheets from different sources. (**a**) B PF sulfite pulp; (**b**) B SF sulfite pulp; (**c**) UB PF kraft pulp; (**d**) UB SF kraft pulp; (**e**) MP fines; (**f**) MFC 1; (**g**) MFC 2; (**h**) MFC 3.

In order to evaluate whether the differences in apparent density can be related to the morphology of the different samples on the micro/nanoscale, the sheets were investigated by AFM in a dry state. Figure 6 depicts the amplitude images (10 × 10 µm^2^). The MFC samples (a–c) show small features in the chosen frame (10 × 10 µm^2^), while in the fines samples, fragments of fibers are visible.

In order to evaluate whether these observations can be determined at a larger scale, profilometry measurements with a Bruker DekTak XT surface profiler (scanning needle dip 12.5 µm) were performed. Table 4 summarizes the findings on the determined RMS roughness of the paper sheets. Therefore, profilometry allows for a differentiated picture of the morphology of the sheets. The MFC derived sheets exhibit RMS values of 1.9 to 2.3 µm, while those from the other materials, with the exception of SF kraft pulp fines, have higher roughness values.

From this data it can be seen that the density of the sheets can be correlated to some extent to their RMS roughness. Besides the MFC sheets, the sheets from B PF sulfite and UB SF kraft pulps feature the lowest RMS roughness among all the prepared fine sheets, and, in addition, they have a comparable density as the MFC sheets.

**Figure 7:**
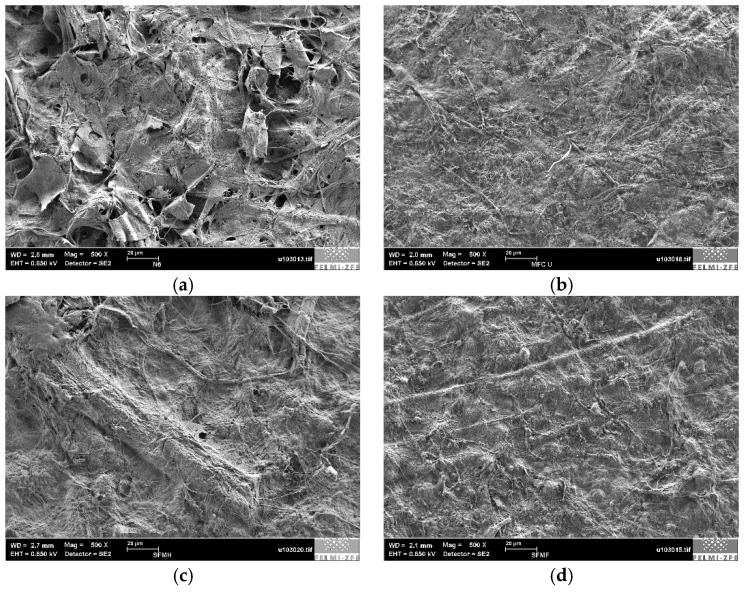
Low voltage scanning electron microscopy (LVSEM) images of sheets from different sources. (**a**) MP fines; (**b**) MFC 2; (**c**) B SF sulfite pulp; (**d**) UB SF kraft pulp.

Selected sheets (MP fines, MFC 2, and secondary fines from kraft and sulfite pulp) were also investigated using low voltage scanning electron microscopy (LVSEM). A rather low magnification of 500× was chosen in order to visualize features in a similar scale to the needle used in profilometer measurements presented in Table 4. The results presented in Figure 7 correlate to the measured RMS data using profilometry.

#### 3.2.3. Mechanical Properties of the Sheets

**Figure 8:**
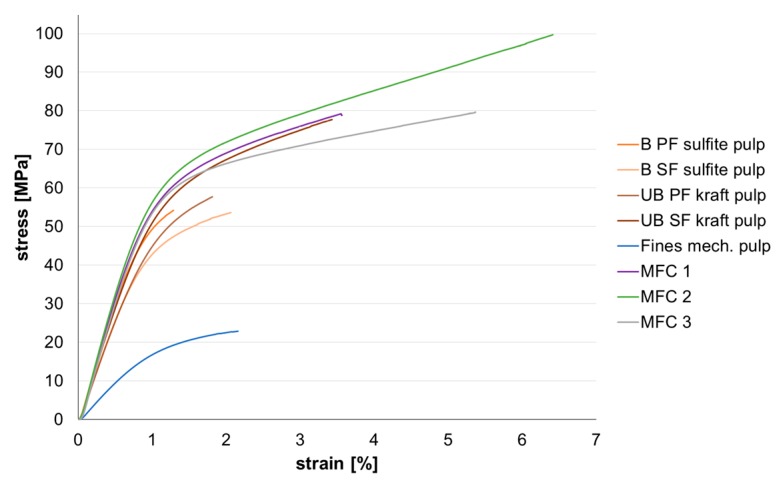
Stress-strain curves of fines and MFC sheets (*v* = 5 mm/min, a free clamping length: 50 mm, strip width: 15 mm).

Figure 8 shows the stress-strain curves obtained from mechanical testing using a Zwick tensile tester. The MFC sheets show the highest stress-strain values, followed by the secondary fines from unbleached kraft pulp. When comparing the results obtained for kraft and sulfite fines, it is apparent that the well-known advantages of kraft pulp over sulfite pulp in strength are also observed when comparing fines sheets. Due to their more fibrillar character, secondary fines show for both pulps a better stress-strain behavior when compared to primary fines, with the difference being more pronounced for the kraft pulp fines. Although sulfite, kraft, and MFC fines show a similar behavior in the linear region, thus similar E-modulus or stiffness, secondary kraft fines and all three MFC samples exhibit more strain before breaking.

Handsheets prepared from the mechanical pulp fines have a significantly lower breaking load and breaking stress when compared to the other materials. This is due to the lower bonding ability due to higher lignin content, as well as the lower sheet density this material achieves (see Figure 5). The lower stress can also partly be attributed to the higher thickness of the mechanical pulp fines sheets, as with thickness, the load bearing area increases leading to lower breaking stresses.

#### 3.2.4. Contact Angle Determinations and Surface Free Energy (SFE)

**Figure 9:**
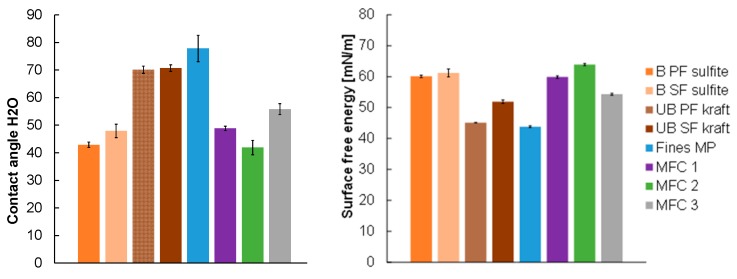
Static water contact angles of the paper sheets (**Left**) and the corresponding surface free energies determined by the Owens-Wendt-Rabel-Kaelble (OWRK)approach (**Right**). As apolar liquid diiodomethane was used.

For all the sheets, static contact angles (SCA) have been determined using water and diiodomethane. It can be clearly seen in Figure 9 that there are major differences in the wettability of the different sheets with water.

As expected, bleaching of the pulp prior to sheet formation increases the hydrophilicity of the paper sheets, mainly due to lignin removal. In fact, the determined Kappa numbers (see Table 2) match very well the trends of the SCA with water, which means that the major part of the lignin is either homogenously distributed in the fiber, or accumulated at the individual fiber surfaces. Consequently, SCA’s with water decrease from ca. 70°, to below 50°. For all of the sheets prepared from MFC’s (MFC 1–3), SCA with water are in this range as well. The same behavior is observed in the SFE where the bleaching step increases the SFE of the sheets corresponding to a higher degree of hydrophilicity.

## 4. Conclusions

Sheet properties of primary and secondary pulp fines from different sources were compared to MFC sheets, together with the use of novel equipment for fines separation in the kg scale from paper pulps. The MFC samples tend to form the densest and smoothest sheets, also achieving the highest strength. Sheets from secondary fines from unbleached kraft pulp exhibit structural and mechanical properties coming close to MFC. The known differences between kraft and sulfite pulps on the fiber level are also observable at the level of fines from these pulps. Sheets formed from mechanical pulp fines exhibit a coarser surface, show the lowest apparent density, and significantly lower stress-strain values. Hydrophilicity of the fines sheets is mainly depending on the chemical composition, with higher lignin content imparting a more hydrophobic character.

Because of their wide spectrum of properties depending on pulp type and their similar character to coarser MFC grades, fiber fines as a by-product of the paper and pulp industry might find applications in various fields where presently the use of MFC grades is evaluated.

## Figures and Tables

**Table 1:** Fines and microfibrillated celluloses (MFC) samples used in this study.

Type of Fines/MFC	Labelling of Samples
Never-dried, bleached sulfite pulp, primary fines	B PF sulfite pulp
Never-dried, bleached sulfite pulp, secondary fines	B SF sulfite pulp
Never-dried, unbleached softwood kraft pulp, primary fines	UB PF kraft pulp
Never-dried, unbleached softwood kraft pulp, secondary fines	UB SF kraft pulp
Bleached pressurized ground wood (PGW), fines	MP fines
MFC 1 (experimental MFC, undisclosed source)	MFC 1
MFC 2 (commercially available MFC A)	MFC 2
MFC 3 (commercially available MFC B)	MFC 3

**Table 2:** κ numbers of the different cellulosic materials.

Header	κ Number
B PF sulfite pulp	3.4
B SF sulfite pulp	2.8
UB PF kraft pulp	75.1
UB SF kraft pulp	50.7
Fines MP	162.1
MFC 1	1.2
MFC 2	0.7
MFC 3	0.6

**Table 3:** Mean particle size of pulp fines (fractionated using the pressure screen) and MFC samples (as provided), measured with the L & W Fiber Tester Plus; resolution 3.3 µm/pixel.

Header	CED_q^2^_ (µm) *
B PF sulfite pulp	40.35
B SF sulfite pulp	34.59
UB PF kraft pulp	52.73
UB SF kraft pulp	33.04
Fines MP	37.16
MFC 1	52.11
MFC 2	38.19
MFC 3	29.84

* CED: circle equivalent diameter.

**Table 4:** RMS roughness of sheets prepared from fines and MFC determined by profilometry and labels used in Figure 6.

Header	RMS Roughness (µm)	Label
B PF sulfite pulp	2.3 ± 0.1	a
B SF sulfite pulp	2.5 ± 0.1	b
UB PF kraft pulp	3.0 ± 0.2	c
UB SF kraft pulp	1.7 ± 0.2	d
Fines MP	3.4 ± 0.3	e
MFC 1	2.3 ± 0.3	f
MFC 2	1.9 ± 0.3	g
MFC 3	2.3 ± 0.1	h
